# The Role of Self-Care Activities (SASS-14) in Depression (PHQ-9): Evidence From Slovakia During the COVID-19 Pandemic

**DOI:** 10.3389/fpubh.2021.803815

**Published:** 2022-01-17

**Authors:** Beata Gavurova, Boris Popesko, Viera Ivankova, Martin Rigelsky

**Affiliations:** ^1^Center for Applied Economic Research, Faculty of Management and Economics, Tomas Bata University in Zlín, Zlín, Czechia; ^2^Department of Business Administration, Faculty of Management and Economics, Tomas Bata University in Zlín, Zlín, Czechia; ^3^Institute of Earth Resources, Faculty of Mining, Ecology, Process Control and Geotechnologies, Technical University of Košice, Košice, Slovakia; ^4^Department of Marketing and International Trade, Faculty of Management and Business, University of Prešov, Prešov, Slovakia

**Keywords:** depression, mental health, health consciousness, nutrition and physical activity, sleep quality, coping strategies, COVID-19, self-care behavior

## Abstract

In the ongoing situation, when the world is dominated by coronavirus disease 2019 (COVID-19), the development of self-care programs appears to be insufficient, while their role in mental health may be crucial. The aim of the study was to evaluate the associations between self-care activities and depression in the general Slovak population, but also in its individual gender and age categories. This was achieved by validating the self-care screening instrument, assessing differences, and evaluating the associations using quantile regression analysis. The final research sample consisted of 806 participants [males: 314 (39%), females: 492 (61%)] and data were collected through an online questionnaire from February 12, 2021 to February 23, 2021. Patient Health Questionnaire (PHQ-9) for depression (α = 0.89) and Self-Care Activities Screening Scale (SASS-14) [health consciousness (HC) (α = 0.82), nutrition and physical activity (NPA) (α = 0.75), sleep quality (SLP) (α = 0.82), and interpersonal and intrapersonal coping strategies (IICS) (α = 0.58)] were used as screening measures. Mild depressive symptoms were found in 229 participants (28.41%), moderate depressive symptoms in 154 participants (19.11%), moderately severe depressive symptoms in 60 participants (7.44%) and severe depressive symptoms in 43 participants (5.33%). The main findings revealed the fact that individual self-care activities were associated with depression. This supported the idea that well-practiced self-care activities should be an immediate part of an individual's life in order to reduce depressive symptoms. Sleep quality played an important role, while HC indicated the need for increased attention. Other dimensions of self-care also showed significant results that should not be overlooked. In terms of depression, females and younger individuals need targeted interventions. The supportive educational intervention developed based on the self-care theory can help manage and maintain mental health during a stressful period, such as the COVID-19 pandemic. Health policy leaders should focus on health-promoting preventive self-care interventions, as the demand for them increases even more during the pandemic.

## Introduction

With the onset of coronavirus disease 2019 (COVID-19), people's daily lives changed within a few days as daily routines were interrupted and people were locked up at home. In this context, the ongoing COVID-19 pandemic represents a health burden not only in terms of the spread of a life-threatening infection, but also serious psychological consequences (1–4). The fear of infection as well as sudden changes in everyday life play a major role in this situation. Many countries have imposed strict measures and restrictions to successfully defeat COVID-19, with lockdown, quarantine, and isolation being the main strategies for victory (5). On the other hand, isolation and social distance are factors that increase the risk of poor mental health (6). Moreover, individuals had to face an unknown disease, worries about transmission, insecurity, but also new realities such as wearing a mask, home office, or home schooling (7, 8). In this way, evidence has shown that people are less able to control critical situations and manage stressful events related to severe acute respiratory syndrome compared to the stressful events of everyday life (9). Based on all these findings, the COVID-19 pandemic can be considered as a global trauma with consequences for mental health (6, 10).

From a mental health perspective, depression is a huge burden on health (11). In Slovakia, together with the COVID-19 pandemic, depressive symptoms also appeared across the population (12, 13), while depression is considered not only a health but also an economic burden in this country (14). In addition, it has been proven that Slovak family members of patients in intensive care units report a higher prevalence of depression (15), which can also be expected in COVID-19 disease. Young people, patients as well as females can be considered as risk and vulnerable groups in this country (16–20). On the other hand, there is little evidence among the general Slovak population, which was confirmed by the results of a new international study conducted by Zhang et al. (21). Although depression is a well-examined problem in Europe (22, 23), Slovakia is a European country that has long overlooked and neglected this serious health problem. There is an obvious insufficiency in the field of research, but also in the field of implementation of prevention and treatment strategies in practice (24). This is reflected in the lack of evidence-based interventions.

Following the above-mentioned facts, it should also be noted that the mental health of the population plays an important role in the success or failure of pandemic management, public policies and health measures to overcome the pandemic, but also in the success of communicating the importance of the measures, vaccination and COVID-19 risks (25). In this context, self-care behavior is considered to be one of the main strategies to eliminate not only the transmission of infection but also the psychological effects of the COVID-19 pandemic (26). Self-care covers a range of activities and approaches that an individual pursues to maintain physical and mental health, as well as to manage ill health (27). In these activities, individuals are encouraged by their self-care abilities, which represent the fundamental pillars of self-care, and by their self-efficacy, which facilitates the acquisition of the desired effects (28). According to Butler et al. (29), there are two objectives of self-care, namely to protect or manage stress and other negative situations, but also to maintain or enhance well-being and overall functioning. The authors also stated six life domains that need attention in terms of self-care activities: physical, professional, relational, emotional, psychological, and spiritual (29).

The lack of research efforts in Slovakia can be observed not only for depression, but also for self-care activities. In other words, this issue as a whole is not adequately researched in Slovakia. There is limited evidence on self-care behavior, while previous studies have focused mainly on professional helpers as a risk population group (30–32). The authors of these studies emphasized that increased and continuous attention is needed to promote the value of self-care behavior in this country. At the same time, they stated that health status plays an important role in self-care behavior (31, 32). The foreign evidence has shown that improvements in physical health, vitality, social functioning, emotions, and mental health can be expected if self-care interventions are involved in individuals' lives (33). Thus, the benefits of self-care activities are unquestionable (34) and their practice can be reflected in increased satisfaction (35). In this way, self-care is an important aspect of health promotion aimed at improving population health and well-being (33, 36). Self-care activities, as part of hygiene practices, are effective in coping with stress and preventing health problems, while the motivation to act and include self-care elements into daily routine plays an important role (37).

Bearing in mind the evidence presented above, it can be assumed that self-care activities are a core of mental health, especially in the stressful period of the COVID-19 pandemic. The main components of the self-care conceptual model take into account health literacy and self-awareness, health consciousness (HC), knowledge, mental well-being, healthy eating, physical activity, good hygiene, and risk avoidance (36, 38). Among these components, sleep quality (SLP) appears to be an important predictor of mental health and well-being, while physical and nutrition activity also plays a significant role (39). In terms of depression, several self-care activities, such as SLP, seemed to be inversely associated with this serious mental disorder (40). In this context, self-care behavior can be considered a predictor of depression (39).

In various countries, the presented issue has been examined mainly in terms of the role of depressive symptoms in self-care activities (41–44), but research area lacks knowledge about the role of self-care activities in depression (39, 40). Thus, this study contributes to addressing the limitations in the current literature by providing a better understanding of the problem. At the same time, international research has largely focused on patients rather than the general population, while the analyzes have covered only some of the activities that fall within the concept of self-care behavior. All these facts were the motivation for the authors of this study, which enriches scientific knowledge as such. It should also be noted that similar research has not yet been carried out in Slovakia. The presented study focuses on the associations between self-care and depression in a non-patient sample with respect to the whole concept of self-service activities. The resulting insights are of great importance for public health in Slovakia, and the findings provide guidance to public health leaders in improving mental health and promoting self-care. This research is particularly needed during the COVID-19 pandemic, which left trauma in the lives of individuals.

## Methodology

The aim of the presented study was to evaluate the associations between self-care activities and depression in the general Slovak population, but also in its individual gender and age categories.

### Measures

The analytical procedures included a four-factor measure related to the concept of self-care, that is Self-Care Activities Screening Scale (SASS-14) (38). This instrument was developed to screen specific self-care activities during the COVID-19 pandemic with regard to HC and consists of the following dimensions (subscales): (i) health consciousness—HC (α = 0.82), (ii) nutrition and physical activity—NPA (α = 0.75), (iii) sleep quality—SLP (α = 0.82), and (iv) interpersonal and intrapersonal coping strategies—IICS (α = 0.58). The SASS-14 items offered possible responses using a 6-point Likert scale (numerical coding): (1) never, (2) very rarely, (3) rarely, (4) occasionally, (5) very frequently, (6) always. The higher the total and subscales scores, the higher the frequency of self-care activities performed by individuals.

The second measure was represented by the Patient Health Questionnaire (PHQ-9) for screening depression (45). This brief instrument in the form of a self-report questionnaire is able to diagnose not only depressive symptoms but also the severity of depression. The PHQ-9 instrument was selected based on its acceptance and common use in the professional and scientific community. The following responses were provided to PHQ-9 items (numerical coding): (1) not at all, (2) several days, (3) more than half the days, (4) nearly every day. The participants' responses recorded the period of the past 2 weeks before completing the questionnaire. The instrument provides a total score ranging from 9 to 36 with thresholds: 14–18 mild depressive symptoms, 19–24 moderate depressive symptoms, 25–29 moderately severe depressive symptoms, >29 severe depressive symptoms. Thus, the higher the total score, the more severe the depression. Cronbach's α was 0.89 (confidence interval—CI: 0.88–0.90).

### Participants and Data Collection

A total of 958 responses were obtained, 152 of which were excluded due to non-compliance with criteria such as approved consent to participate in the survey, age over 18 years, but also due to system error, incomplete data, and irrelevant responses. Thus, 806 participants were included in the final research sample. In addition to screening measures presented above, the questionnaire also collected various socio-demographic information about participants. In terms of gender, there were 314 males and 492 females. Age was expressed using generational categories: participants born before 1980 (>41 years) = 176, between 1980 and 1989 (32–41 years) = 113, between 1990 and 1999 (22–31 years) = 427, in 2000, and later (<22 years) = 90. Females and young adults were slightly predominant in the research sample, but this limitation should not be considered as a bias that could significantly impair the results. In terms of social status, students slightly predominated (full-time student = 364, pensioner (old-age, disabled, etc.) = 26, maternity leave/guardianship = 18, unemployed = 31, entrepreneur = 50, employed = 317).

Data were collected through an online questionnaire from February 12, 2021 to February 23, 2021. Thus, the collection took 12 days, which can be considered a strength of research, as possible externalities during the pandemic with changing conditions were minimized. The subjects were the adult Slovak population. The data collection process was based on quota selection respecting gender, age and social status. The effort was to achieve a proportionally divided sample by gender. In terms of social status, a maximum of 30% of students, 50% of workers, and a maximum of 20% of other categories were expected. In terms of age, it was expected that 10% of participants were born in 2000 and later, while in the other three categories there was an effort to achieve approximately proportional representation. Some deviations from the country population could be observed, i.e., young people, females and students predominated. This can be considered a limitation of the study. On the other hand, the data collection was completed after 12 days as planned, because the risk of skewing results due to external social influences was more severe than the risk of some deficiencies in the sample. The time of collection was considered to be the most serious attribute of the negative effects on the sample during the pandemic.

The questionnaire was freely shared, but also promoted on the social network Facebook, while the target audience was controlled. Subsequently, the questionnaire was distributed to groups on the social network with a specific request for completion. Similar requests were sent by emails, which were obtained from publicly available databases.

### Governance and Ethics

The study was conducted according to the guidelines of the Declaration of Helsinki (46). The research was approved by the Ethics Committee of the Clinical Trials Services, USP TECHNICOM, Technical University of Košice, Slovakia (Ref. 02/03/2021 IG Bioinformatics). At the beginning of the questionnaire, all participants received the same information about the research and they were provided with information about their rights and anonymity. All participants included in the research confirmed their informed consent. The participants did not receive any financial reward.

### Statistical Analysis

The following statistical approach was selected to meet the main aim of this study. The characteristics of the central tendency (mean, median) were used for the statistical description. The level of reliability was verified by Cronbach's α. Non-parametric tests of differences (Wilcoxon signed-rank test, Kruskal Wallis test) were applied to evaluate possible differences in self-care activities and depression between individual population categories. The preference for non-parametric statistical methods was conditioned by the fact that several variables or groups of variables did not meet the conditions for the use of parametric tests (normality, homogeneity of variances). Correspondence analysis was performed using Pearson's χ^2^-test. Finally, the associations between self-care activities and depression were verified using quantile regression (Percentile: λ = 0.25, 0.50, 0.75). Quantile regression analysis was preferred over other regression models, as this method is able to minimize the risk of skewing results due to identified deficiencies in the sample (deviations from the population).

The analytical calculations were performed using the programming language R v 4.1.1 (RStudio, Inc., Boston, MA, USA) and SPSS v 26 (Armonk, NY: IBM Corp.).

## Results

This section presents the main results and their interpretation. The results were obtained through several analytical procedures, including a statistical evaluation of the validity of the SASS-14 instrument, an assessment of the differences in the measured scores between gender and age categories, as well as a statistical examination of the associations between self-care activities and depression. At the beginning, a description analysis and a difference analysis were performed in order to provide a more detailed view of the analyzed data. Subsequently, a correspondence analysis focused on the links between gender-age characteristics, self-care activities in selected dimensions, and depression. At the end of this section, the main results of a quantile regression analysis were offered to determine the associations between self-care activities and depression.

Table 1 provides an overview of the latent variables (LV), which consist of manifest variables (MV) with the relevant identification number (ID), as well as their full wording. These LVs were included in the subsequent analyzes and were formed by the arithmetic mean of the individual MVs of the SASS-14 instrument and the sum of the PHQ-9 instrument. The measures of central tendency (mean, median) are offered for individual items of the questionnaire.

**Table 1:** Description of the data.

**LV ID**	**MV ID**	**Questionnaire item**	**Mean**	**Median**	**Cr α (CI)**
HC	HC 1	I am alert to changes in my health	4.84	5	0.82
	HC 2	I am usually aware of my health	5.25	5	(0.81–0.84)
	HC 3	I reflect about my health a lot	4.35	5	
	HC 4	I know my inner feelings about my health	4.95	5	
	HC 5	I am constantly examining my health	3.73	4	
NPA	NPA 1	I do physical activity (some sport, yoga, or dance) for at least 30 min a day	4.06	4	0.75
	NPA 2	I eat three servings of fruit and two of vegetables daily	4.36	5	(0.72–0.75)
	NPA 3	I think I am eating better than I used to (less sugar, salt, fried snacks, or precooked food)	4.06	4	
	NPA 4	I'm drinking an average of eight glasses of water a day	4.56	5	
SLP	SLP 1	I sleep 7–8 h a day	4.68	5	0.82
	SLP 2	I think that my rest is of quality	4.41	5	(0.79–0.84)
IICS	IICS 1	I am learning to do new things like: playing an instrument, sports, practicing a new language, cooking, painting, new apps, video games, etc.	3.80	4	0.58
	IICS 2	I actively participate in the initiatives of my community (e.g., clapping, singing, playing music, offering my support in what I could help, etc.)	2.53	2	(0.35–0.63)
	IICS 3	I am finding moments to be more connected to myself (I observe, write, or reflect on my thoughts, emotions, or behaviors)	4.25	4	
PHQ-9	PHQ-9 1	Little interest or pleasure in doing things	2.14	2	0.89
	PHQ-9 2	Feeling down, depressed, or hopeless	2.02	2	(0.88–0.90)
	PHQ-9 3	Trouble falling or staying asleep, or sleeping too much	1.92	2	
	PHQ-9 4	Feeling tired or having little energy	2.33	2	
	PHQ-9 5	Poor appetite or overeating	1.81	1	
	PHQ-9 6	Feeling bad about yourself—or that you are a failure or have let yourself or your family down	1.74	1	
	PHQ-9 7	Trouble concentrating on things, such as reading the newspaper or watching television	1.95	2	
	PHQ-9 8	Moving or speaking so slowly that other people could have noticed? Or the opposite —being so fidgety or restless that you have been moving around a lot more than usual	1.32	1	
	PHQ-9 9	Thoughts that you would be better off dead of or hurting yourself in some way	1.37	1	

*LV, latent variable; MV, manifest variable, Cr α, Cronbach's α; CI, confidence interval; HC, health consciousness; NPA, nutrition and physical activity; SLP, sleep quality; IICS, interpersonal and intrapersonal coping strategies; PHQ-9, patient health questionnaire*.

As stated in the methodology, the SASS-14 questionnaire items were scored in the interval 1 (never) to 6 (always), which means that the higher the number, the more frequent the specific self-care activity. In general, the mean and median values of the self-care activities ranged from 4 to 5 (Table 1). This finding revealed the fact that Slovak participants performed individual self-care activities occasionally or very frequently during the COVID-19 pandemic. The only exception was participation in the initiatives of participants' community (IICS 2), which was very rare among participants (mean = 2.53; median = 2). On the other hand, health awareness was very frequent among participants (HC 2: mean = 5.25). Self-care behaviors such as alertness to changes in health (HC 1) or knowledge of inner feelings about health (HC 4) were also frequent. The PHQ-9 questionnaire items for depression were scored from 1 (not at all) to 4 (nearly every day). As can be seen, the mean values ranged from 1.32 to 2.33, indicating that Slovak participants reported individual depressive symptoms in several days during the past 2 weeks.

Based on the values of Cronbach's α, the reliability level could be considered acceptable in almost all cases analyzed. Only an item concerning IICS proved to be weaker in terms of reliability, and this could be considered as a certain limitation of the research.

**Figure 1:**
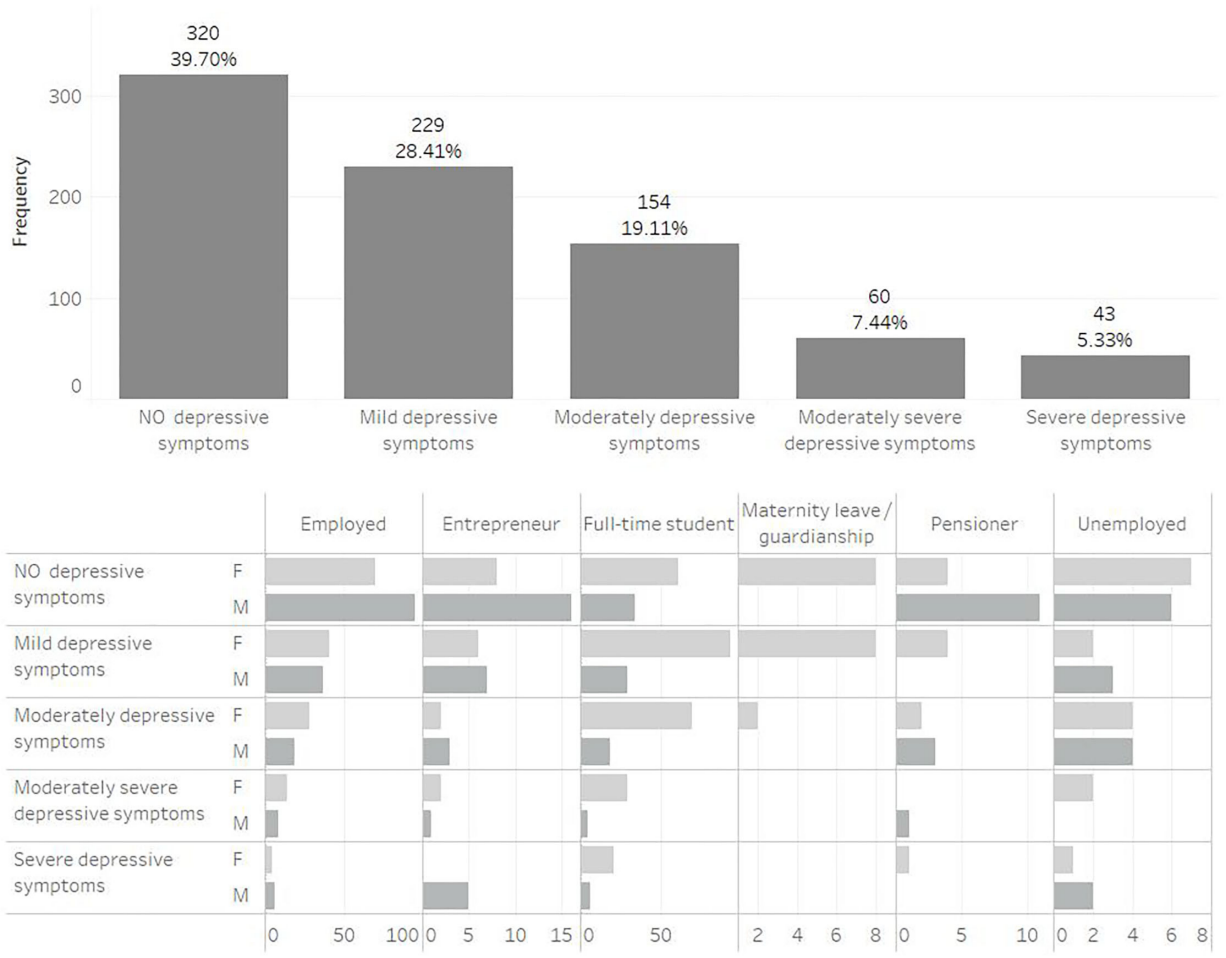
Frequency of depressive symptoms among Slovak participants.

Figure 1 provides more detailed information on depressive symptoms in Slovakia, while participants were assigned to one of five categories based on their depression score (PHQ-9). As can be seen, no depressive symptoms were found in 320 participants (39.70%). On the other hand, 229 participants (28.41%) reported mild depressive symptoms, 154 participants (19.11%) reported moderate depressive symptoms, 60 participants (7.44%) reported moderately severe depressive symptoms and 43 participants (5.33%) reported severe depressive symptoms. The results are also presented in terms of social status.

The following analyzes included the average scores of individual self-care activities (HC, NPA, SLP, and IICS) and the depression score (PHQ-9) as the sum of the values in the individual items. This approach was in line with the recommended procedure for adjusting selected scales.

**Figure 2:**
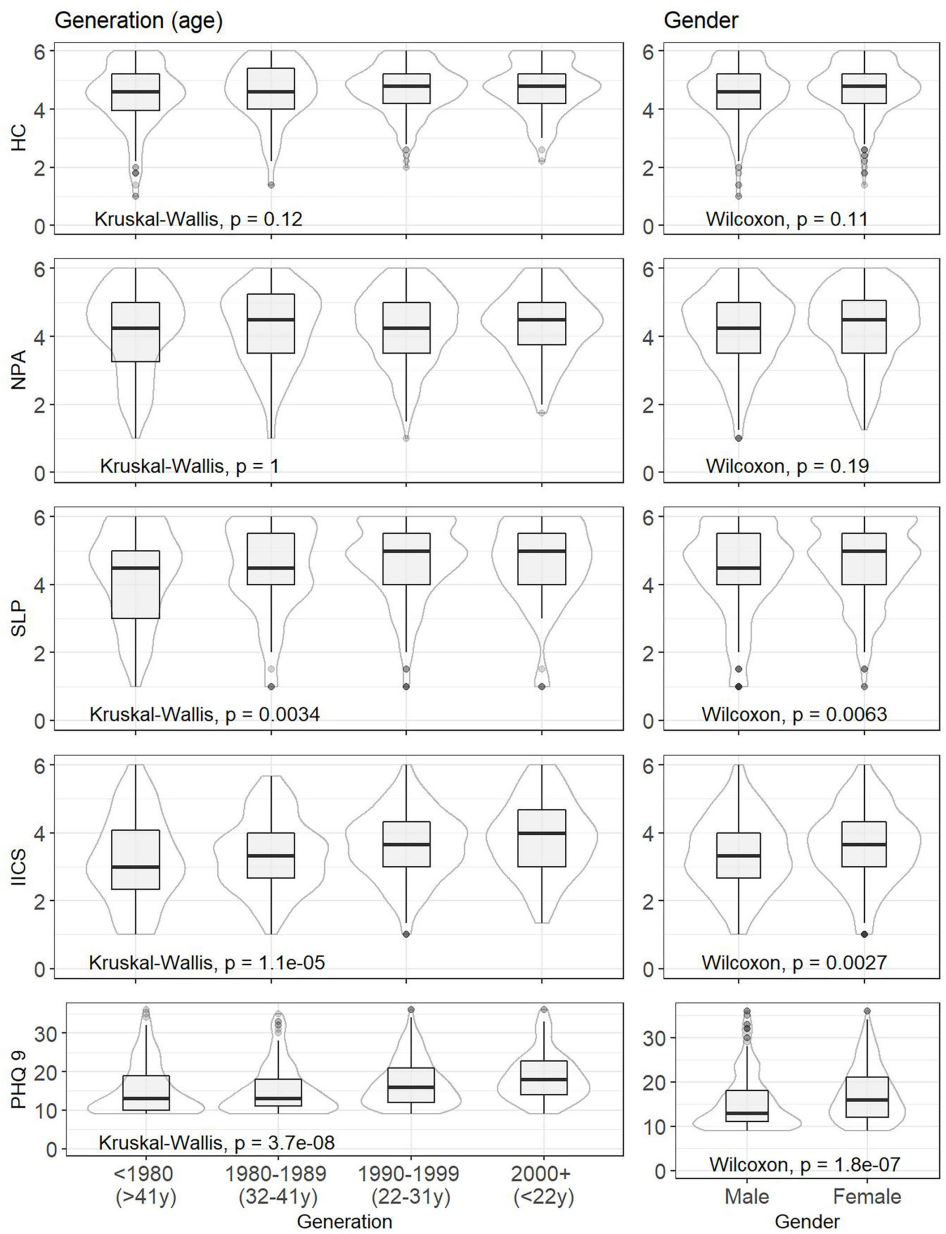
Selected statistical characteristics of indicators and results of difference tests—classification by age and gender.

Figure 2 shows self-care activities and depression in box plots, as well as the results of difference tests. This allows a closer look at the examined indicators. On this basis, significant differences between individual age categories and between gender categories were found in SLP, IICS, and depression (PHQ-9). This justifies the idea of examining the associations between self-care activities and depression in age and gender classifications. In terms of gender, females reported significantly higher levels of depression than males. Females also reported more self-care activities such as IICS and SLP. From an age perspective, younger participants were more prone to depression, and they reported more self-care activities such as IICS and SLP. Accordingly, significantly less IICS and SLP were observed among older participants aged 32 years and over (age categories: >41 years, 32–41 years).

Figure 2 also points to the median values of the indicators in individual population groups. The median value of 15 was found for all participants, which means mild depressive symptoms. Mild depressive symptoms were also common for females (median = 16), but not for males (median = 13). The youngest participants reported mild depressive symptoms, but their median score was on the verge of mild and moderate depression (median = 18). This was not the case for the oldest participants (median = 13).

**Figure 3:**
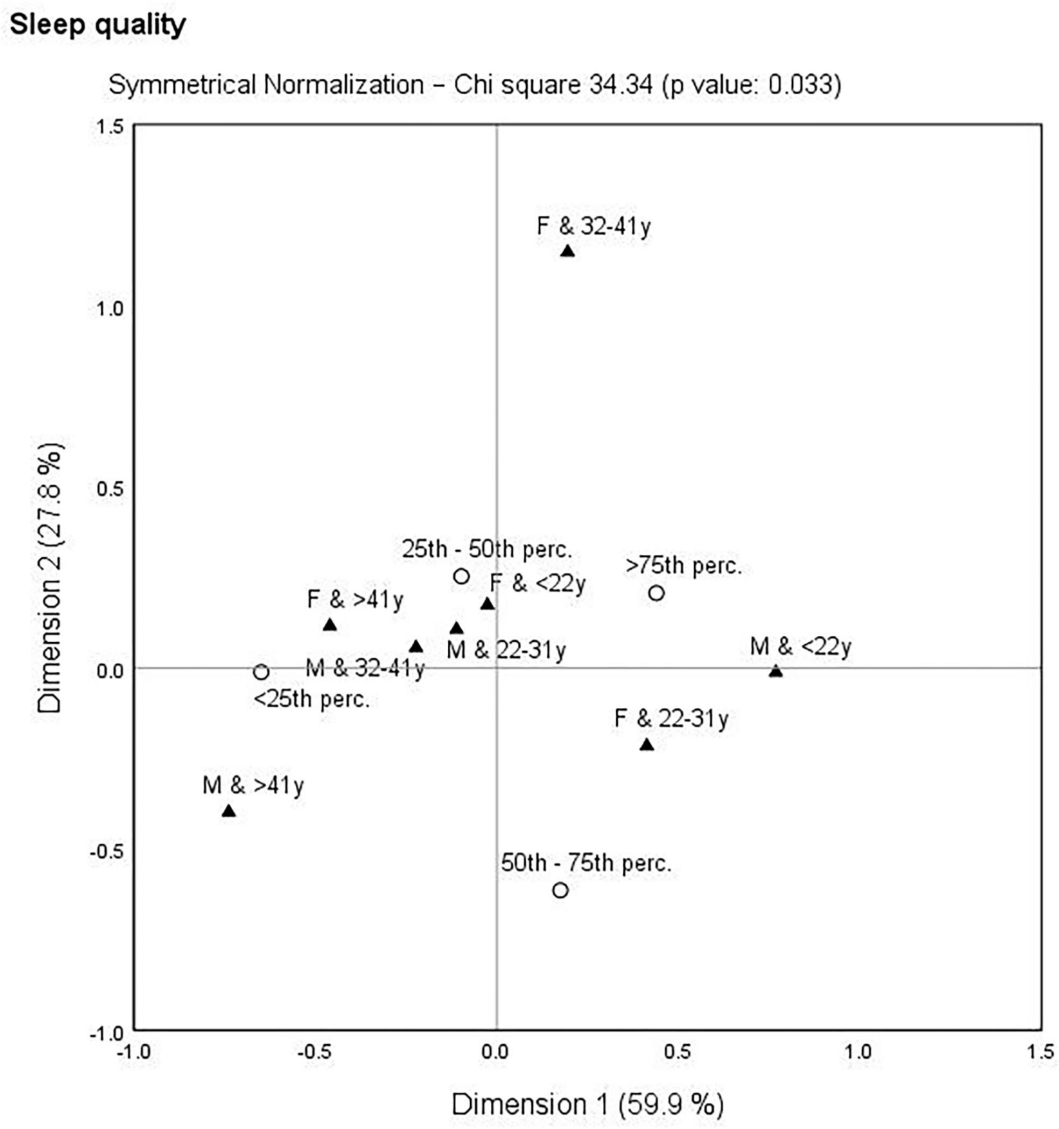
Correspondence map—sleep quality (SLP) and gender-age characteristics.

**Figure 4:**
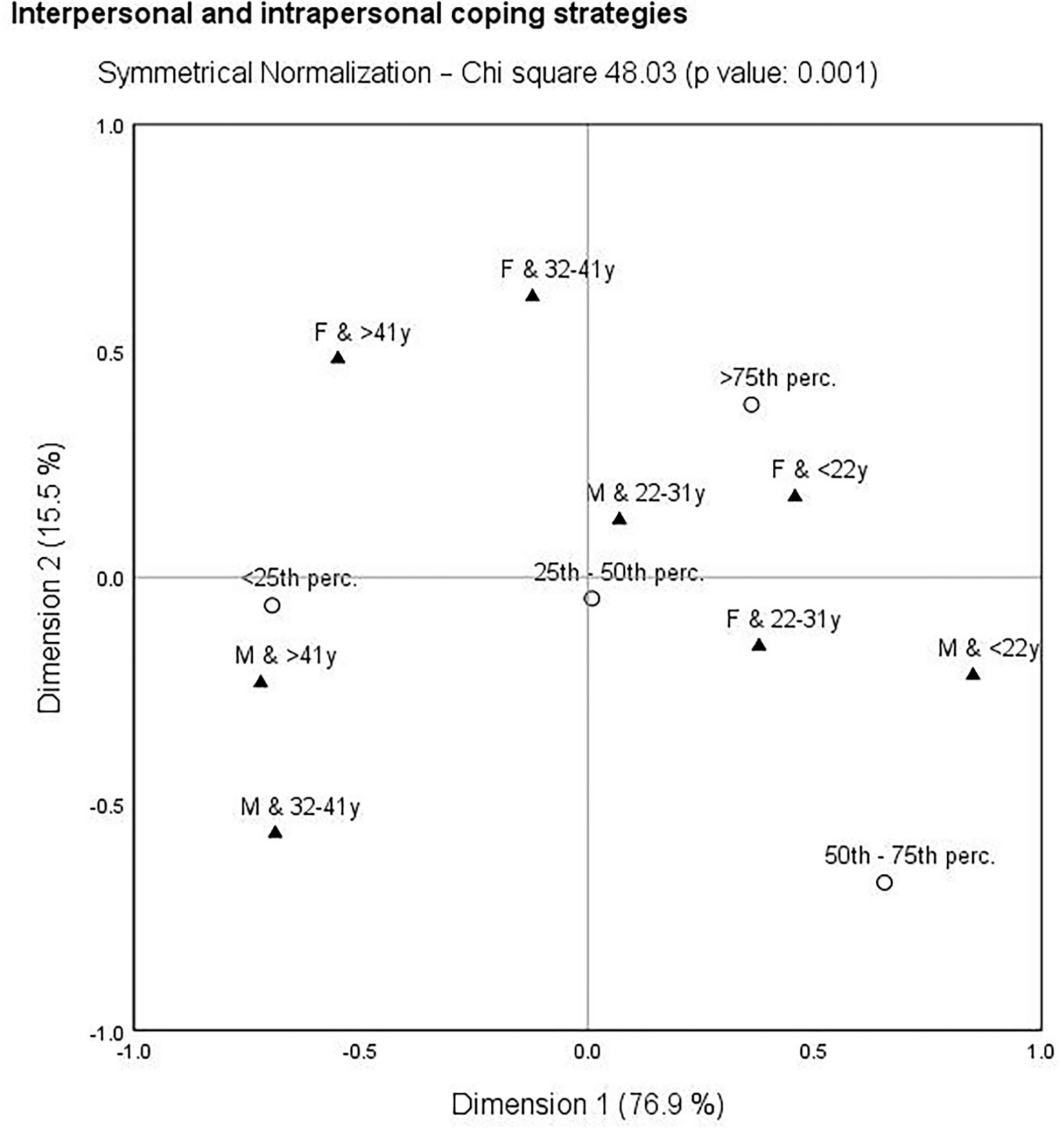
Correspondence map—interpersonal and intrapersonal coping strategies (IICS) and gender-age characteristics.

**Figure 5:**
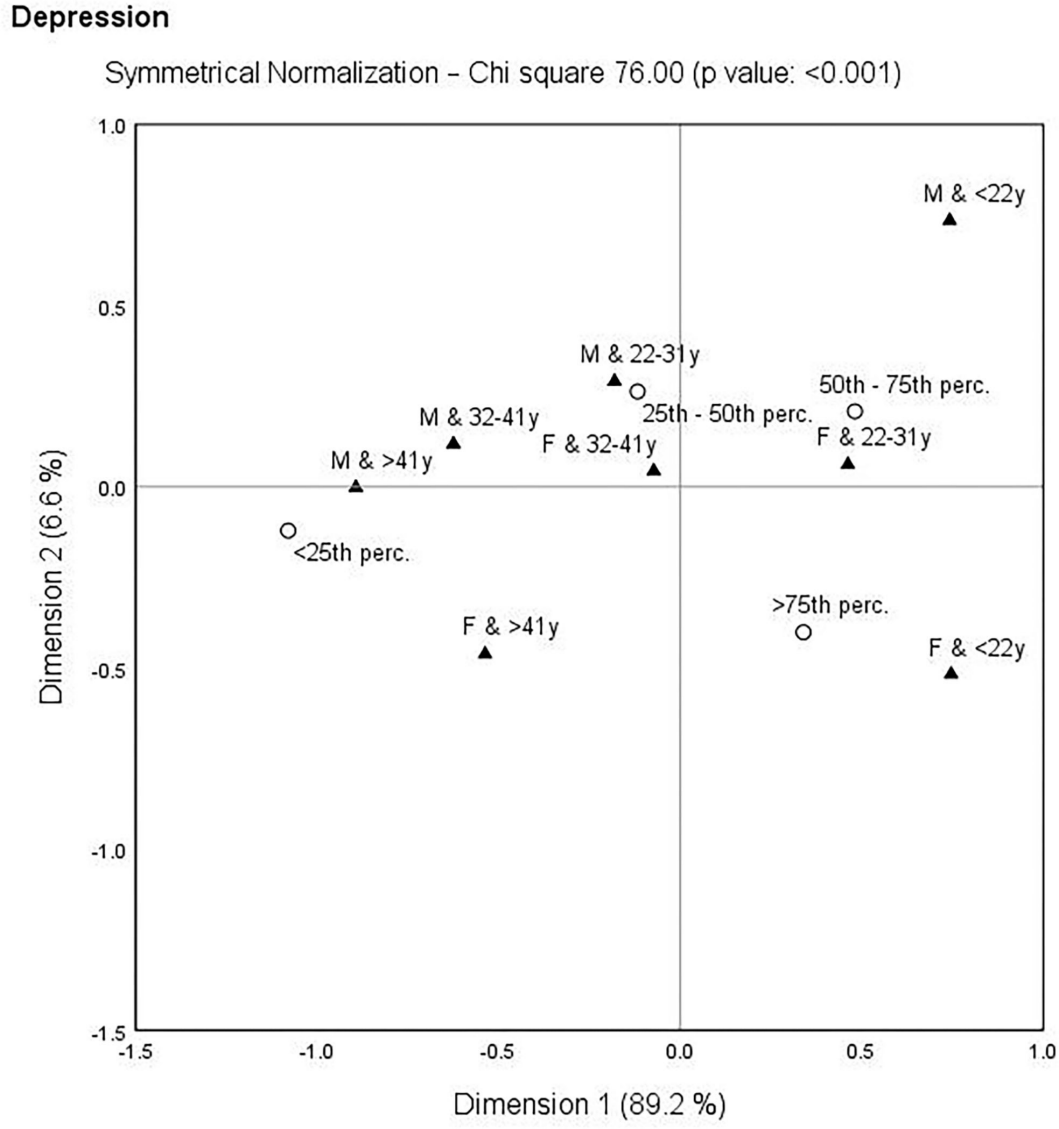
Correspondence map—depression (PHQ-9) and gender-age characteristics.

The following correspondence analysis was used to assess the links between self-care activities, depression, and gender-age characteristics. The identification of the closest links can be important from a public health point of view, as it more precisely defines the population group to which increased attention should be paid. Self-care and depression indicators were transformed into percentiles (<25th perc., 25th−50th perc., 50th−75th perc., >75th perc.) and gender-age categories were merged (oldest males: M and >41 years, older males: M and 32–41 years, younger males: M and 22–31 years, youngest males: M and <22 years, oldest females: F and >41 years, older females: F and 32–41 years, younger females: F and 22–31 years, youngest females: F and <22 years). Based on the results, there was no significant link in terms of HC (χ^2^ = 23.89, *p*-value = 0.298) and NPA (χ^2^ = 15.41, *p*-value = 0.802). In contrast, significant links with gender-age characteristic were identified for SLP (χ^2^ = 34.34, *p*-value = 0.033), IICS (χ^2^ = 48.03, *p*-value = 0.001), and depression (PHQ-9: χ^2^ = 76.00, *p*-value = < 0.001). These links are shown in Figures 3–**5**.

With a focus on Figure 3, which is devoted to SLP and gender-age characteristics, several links could be observed. It is clear that younger participants showed higher SLP compared to older participants. In other words, younger participants were concentrated around the higher SLP. It is also evident that females aged 32–41 years appeared as a distant group.

Figure 4 deals with IICS and gender-age characteristics. It was possible to identify closer links than in the previous figure. The three closest links were found, namely the oldest males (>41 years) were concentrated around the lowest IICS (<25th perc.), younger males (22–31 years) were concentrated around the moderate IICS (25th−50th perc.), and the youngest females (<22 years) were concentrated around the highest IICS (>75th perc.).

Finally, the closest links were observed in Figure 5, which deals with depression (PHQ-9) and gender–age characteristics. It was possible to highlight the link of the oldest males (>41 years) with the lowest depression (<25th perc.), but also the link of the youngest females (<22 years) with the highest depression (>75th perc.).

The purpose of the following quantile regression analysis was to evaluate the associations between self-care activities and depression. In this analysis, depression, as a dependent variable, was divided into quartiles (25th percentile, 50th percentile, 75th percentile). Prior to the application of the analysis, the assumption of multicollinearity was evaluated, while the value of the variance inflation factor did not exceed the limit value of 10 in any of the analyzed cases.

Based on the results of the quantile regression analysis shown in Table 2, several significant associations could be confirmed. For all participants, IICS and HC were positively associated with the lowest depression (λ = 0.25). There were negative associations between SLP and the lowest depression in all participants, males and females. For females, it was also possible to observe that HC was positively associated with the lowest depression.

**Table 2:** Quantile regression analysis—associations between self-care activities and depression for all participants and their categories by age and gender.

**Coef**	**All**	** <1980**	**1980–1989**	**1990–1999**	**2000+**	**Males**	**Females**
		**(>41 years)**	**(32–41 years)**	**(22–31 years)**	**(<22 years)**		
λ = 0.25							
(Intercept)	**10.34**^†^ **(1.36)**	**9.45**^†^ **(2.72)**	12.08^**^(4.74)	2.59^**^(4.88)	16.07^***^(5.97)	**10.8**^†^ **(2.15)**	**11.61**^†^ **(2.02)**
IICS	0.57^**^(0.26)	0.2 (0.63)	0.22 (0.75)	0.41 (1.18)	0.52 (1.02)	0.25 (0.41)	0.63^*^ (0.36)
HC	1^***^(0.31)	0.65 (0.74)	0.59 (0.83)	0.57 (1.07)	1.12 (1.36)	0.59 (0.47)	1.48^***^(0.48)
NPA	−0.31 (0.26)	−0.13 (0.7)	0.2 (1.02)	0.39 (−0.3)	−0.88 (1.13)	−0.05 (0.42)	−0.21 (0.38)
SLP	–**0.86**^†^ **(0.25)**	−0.53 (0.65)	−1.18 (0.76)	0.42 (−2.23)	−1.13 (0.8)	–0.75^**^(0.37)	–**1.53**^†^ **(0.35)**
Pseudo *R*^2^	0.038	0.035	0.052	0.021	0.058	0.033	0.055
λ = 0.5							
(Intercept)	**15.59**^†^ **(1.89)**	9.65^***^(3.31)	17.34^***^(6.36)	3.15^***^(5.62)	16.35^**^(7.01)	**11.84**^†^ **(2.64)**	**15.24**^†^ **(2.5)**
IICS	0.58^*^ (0.3)	−0.13 (0.72)	0.73 (0.99)	0.45 (1.48)	0.52 (1.21)	0.36 (0.47)	0.83^*^ (0.43)
HC	**1.49**^†^ **(0.39)**	1.62^*^ (0.89)	0.92 (1.07)	0.62 (1.99)	1.46 (1.54)	0.9 (0.58)	1.78^***^(0.54)
NPA	−0.57^*^ (0.33)	0.55 (0.81)	−1 (1.25)	0.45 (−0.59)	−0.94 (1.39)	−0.28 (0.51)	−0.58 (0.42)
SLP	–**1.5**^†^ **(0.28)**	–1.37^**^(0.67)	−1.31 (0.87)	0.42 (−4.39)	−0.78 (0.97)	−0.67 (0.43)	–**1.69**^†^ **(0.36)**
Pseudo *R*^2^	0.042	0.039	0.092	0.046	0.071	0.028	0.069
λ = 0.75							
(Intercept)	**21.22**^†^ **(2.47)**	**16.71**^†^ **(3.73)**	**33.26**^†^ **(5.73)**	**3.49**^†^ **(7.49)**	16.26^**^(6.77)	**21.52**^†^ **(4.02)**	**19.2**^†^ **(2.94)**
IICS	0.56 (0.42)	0.93 (0.87)	0.77 (0.94)	0.55 (0.1)	−0.23 (1.2)	0.31 (0.58)	0.61 (0.56)
HC	**1.63**^†^ **(0.49)**	1.61 (1.02)	0.74 (1.07)	0.66 (1.44)	3.08^**^(1.48)	1.13 (0.73)	**2.29**^†^ **(0.6)**
NPA	−0.69^*^ (0.41)	0.19 (0.94)	–**3.95**^†^ **(1.14)**	**0.54**^†^ **(0.07)**	−0.56 (1.45)	−0.6 (0.8)	−0.33 (0.48)
SLP	–**1.74**^†^ **(0.33)**	–2.1^**^(0.84)	−0.76 (0.82)	0.43 (−5.36)	−1.07 (1.01)	–1.56^***^(0.58)	–**2.15**^†^ **(0.42)**
Pseudo *R*^2^	0.057	0.054	0.186	0.062	0.069	0.057	0.066

*HC, health consciousness; NPA, nutrition and physical activity; SLP, sleep quality; IICS, interpersonal and intrapersonal coping strategies; PHQ-9: patient health questionnaire.*

*Significant results are highlighted in bold.*

* *p-value < 0.1.*

** *p-value < 0.05.*

*** *p-Value < 0.01.*

† *p-value < 0.001*.

Consequently, SLP was negatively associated with moderate depression (λ = 0.50) in all participants, the oldest participants (>41 years) and females. Also, a significant positive association between HC and moderate depression was observed in all participants and females.

In terms of the highest depression rates (λ = 0.75), a significant association was confirmed in each category of participants. For all participants, the youngest participants (<22 years) and females, HC was positively associated with the highest depression. A significant negative association between SLP and the highest depression was identified for all participants, the oldest participants (>41 years), males and females. Interestingly, NPA was negatively associated with the highest depression in participants aged 32–41 years, while a positive association was observed in participants aged 22–31 years.

The above-mentioned associations could be summarized and interpreted as follows. More IICS were associated with more depression in all participants with the lowest depression score. Higher HC was associated with more depression, especially in all participants and females. More NPA was associated with less depression in people aged 32–41 years, but with more depression in people aged 22–31 years. Higher SLP was associated with less depression, especially for all participants and females.

## Discussion

This study contributes to the issue of self-care and mental health, which has an important position in social and professional discussions, especially during the COVID-19 pandemic. Based on the results, it can be concluded that Slovak participants performed self-care activities occasionally or very frequently during the COVID-19 pandemic. This can be considered a positive aspect during the COVID-19 pandemic, as self-care behavior is very beneficial in the lives of individuals (29, 34). From a public health perspective, it is important that individuals take care of themselves, especially during a difficult pandemic period. Among Slovak participants, health awareness appeared to be a very frequent self-care behavior. Overall, HC was the area of self-care that showed the highest scores. The key message of this finding is that individuals were heavily focused on their health during the health crisis. This can be further supported by public health interventions in such a way that it becomes an integral part of their lives, not only in a crisis situation. On the other hand, participation in community initiatives was very rare. This means that Slovaks did not engage in activities such as clapping, singing, playing music from home, which were popular in other countries during the pandemic. This indicated the diversity of cultures that should be taken into account when creating targeted health-promoting self-care programs. The youngest participants and females reported significantly more self-care activities, especially in terms of IICS and SLP. Focusing on depression, Slovak participants reported individual depressive symptoms for several days during the past 2 weeks. In other words, all participants reported mild depressive symptoms. For public health professionals, this means the need for increased attention and constant monitoring of mental health. Females and young people were the most vulnerable group in terms of depression, and these population groups need increased attention from policy makers when developing successful mental health strategies. These findings support an interesting fact that females and young people were at higher risk of depression despite their higher levels of SLP and IICS. On the other hand, vulnerability of females and young people to psychological symptoms (including depression) during the COVID-19 pandemic was also demonstrated in many other studies (26, 47–50). Xiong et al. (10) also confirmed that common risk factors for mental discomfort during the pandemic were female gender, younger age (under 40 years), but also chronic or psychiatric disease and frequent exposure to social media and news concerning COVID-19. Using correspondence analysis, this study supported that participants' gender-age characteristics were linked with IICS, SLP, and depression. Therefore, gender and age should be taken into account when developing targeted public health strategies. The results agreed with the above-mentioned findings, thus more depression and self-care activities were observed in younger people, while lower scores were found in older people.

This study revealed the fact that several self-care activities were significantly associated with depression. Di Benedetto et al. (40) also emphasized that individuals with the healthiest self-care behaviors were also characterized by the lowest levels of depression. Daniali et al. (44) also revealed a significant association between depression and self-care behavior among Iranian patients with chronic diseases. The opposite perspective was examined among patients with diabetes in a study conducted by Chan et al. (41), who revealed that depression was associated with self-care activities, such as lower rates of reduced or stopped smoking and drinking, less exercise, less regular lifestyle, but also more use of health care and higher rates of foot care. Similar results were confirmed by Chen et al. (42), who found that self-care behaviors affected life satisfaction, while depression affected self-care behaviors and life satisfaction. This evidence confirmed the fact that depression is indirectly and directly associated with self-care (42, 43). The study supports the idea that self-care plays an important role in mental health. This is the key idea that public health professionals should focus on in order to improve the mental health of the population.

Specifically, higher HC was associated with higher depression in all participants (without classification) and females, regardless of depression score, but also in the youngest people (<22 years) with the highest depression score. This can be explained by the fact that those who paid more attention to their health during the COVID-19 pandemic also reported more depressive symptoms. It is well-known that emotional attention is positively related to perceived mental discomfort (51). In other words, individuals with greater concerns about their health may be sensitive to depression during a serious situation such as the COVID-19 pandemic (52, 53). The intensity of worried thoughts and health concerns about COVID-19 were found to be positively correlated with anxiety and depression, and negatively with SLP (54). In terms of the findings revealed in this study, Lee (55) also found that HC is positively related to fear and anxiety and not related to information seeking. According to the authors, health-conscious individuals were more likely to experience mental discomfort than those with low HC. In the context of this study, it is necessary to consider the effect of the pandemic on individuals and what information individuals had or what sources of information they sought. If this information caused health concerns during the pandemic, a higher rate of depression is understandable. Public health efforts should focus on eliminating disruptive information that could adversely affect HC. At this point, health literacy among the population should be underlined (43, 56). According to Wang et al. (57), health literacy has a multiple mediating effect on the relationship between depression and self-care behavior. Therefore, it is important to know what information individuals have and how this information shapes their behavior, mental state and frailty, especially during the COVID-19 pandemic. Health literacy and access to health information are known to improve quality of life (58), but the right information should be provided and communicated in an appropriate way.

It was also found that more NPA was associated with less depression in people aged 32–41 years, but with more depression in people aged 22–31 years. This discrepancy needs to be examined, as evidence from many studies has shown that physical activity and healthier eating habits predict better well-being (59, 60) and lower rates of depression (61–63). In this context, a reduction in exercise duration was considered a risk factor for depression, while an increase in exercise frequency was found to be a protective factor against depressed mood (64). Thus, the promotion of health activities is welcome (65, 66). Some inconsistencies could be observed in healthy eating, as some studies have supported the significant relationship between healthy nutrition and depression (67), while others have not (44). This indicates that NPA is a complex component of self-care and that further deeper investigation is needed to address these discrepancies. The type of questionnaire should also be considered.

Again, interestingly, this study showed that more IICS were associated with more depression in individuals with the lowest depression score. The opposite view was presented by Lara et al. (68), whose results indicated that active coping strategies may be helpful in the management of negative mental states during the COVID-19 pandemic. Miklowitz (69) also stated that cognitive and interpersonal coping strategies are effective for depressive symptoms. Thus, the findings in this study showed some inconsistency with previous findings, which encourages further investigation.

Regarding the quality of sleep, the findings were in line with well-known facts. Accordingly, higher SLP was associated with more depression, especially for all participants (without classification) and females regardless of depression score, for males with the lowest and highest depression score, and for people aged 41 years and over with the highest and moderate depression score. This finding indicated that less depression could be expected with higher SLP, and the opposite view suggested that lower SLP may lead to more depression. In this context, it was possible to support the idea that good SLP is inversely associated with higher levels of depression (40). In contrast, poor SLP can be considered one of the most significant risk factors for mood disorders during the COVID-19 pandemic (70). Lee et al. (71) also emphasized that individuals with poor SLP are more likely to have some or severe problems not only with depression or anxiety, but also with physical activity, self-control and daily activity, and this may be reflected in an impaired quality of life. Thus, it can be concluded that SLP significantly predicts the severity of depressive symptoms (39, 72), and the presented study enriches this knowledge.

In conclusion, the internal consistency of the SASS-14 measure was good with acceptable to high (0.58–0.82) reliability in its subscales, which is in line with the results of the authors of this screening measure (38). The applied tools for measuring depression and self-care activities proved to be reliable for their use in the Slovak population by researchers and experts working in public health.

### Public Health Implications

The findings revealed in this study emphasize the importance of a proactive approach to self-care and the integration of self-care behavior into mental health programs that respect gender and age differences. It is recommended to develop and implement programs to improve self-care behavior across the entire Slovak population, not just patients. The supportive educational intervention developed based on the self-care theory can help manage and maintain mental health not only during a stressful period, such as the COVID-19 pandemic. These programs should focus on increasing and maintaining motivation to practice and include self-care activities in daily routines. This effort would be positively reflected in public health outcomes, as higher levels of self-care knowledge, motivation and skills are expected (73). Gender and age should also be taken into account when developing public health programs aimed at self-care behavior and mental health. In terms of poor mental health, females and younger individuals need targeted interventions. Above all, self-care requires a commitment to an individual's own well-being as a priority (29). In this context, efforts to improve self-care behavior may be more effective if depression is also effectively managed (74).

As the study revealed a positive association between HC and depression, increased attention during the COVID-19 pandemic should be focused on information that shapes HC. One possible explanation for this result could be the high exposure to information about COVID-19, which grows into constant exposure to overwhelming news headlines and misinformation (26, 75). Therefore, in an effort to improve self-service behavior and mental health, emphasis should be placed on the reliability and clarity of information, accessibility, careful communication, and relevant resources. Given the links between health literacy and self-care, health literacy also has a justified place in this problem. A higher health literacy is significantly correlated with greater self-care behavior (76, 77). In addition, health literacy is considered a mediating variable between depression and self-care (57). Therefore, public health leaders should take steps to increase health literacy.

Health-promoting preventive self-care interventions are promising to increase the well-being of healthy individuals (78), and the demand for them increases even more during the pandemic. In the current situation, when the world is dominated by COVID-19, the development of self-care programs in Slovakia appears to be insufficient, but their role in the mental health of the population may be crucial. Despite the importance of this issue, it is still a poorly examined problem. Also, at the level of Slovak public policies, not enough attention is paid to this issue. Expanding the knowledge base would help speed up the process of efforts to implement successful evidence-based strategies. It is therefore appropriate to encourage international cooperation in order to create a valuable information platform, which should then be applied at policy level (79).

### Strengths and Limitations

The study enriches the knowledge base about self-care behavior and its relation to mental health. Thus, this study clarifies the associations between self-care activities and depression in the Slovak population, while respecting gender and age characteristics. As previous literature has focused on the role of depression in individual self-care activities, the results of this study provide novelty in terms of the role of self-care activities in depression. In addition, the research covered the whole concept of self-care and respected gender and age differentiation. The fact that the study is focused on a non-patient sample can also be considered a strength. The findings are of great importance for public health and offer guidance to Slovak public health leaders in terms of improving mental health. Last but not least, this study is an important appeal for the development of health-promoting preventive self-care programs, which are lacking in Slovakia.

Despite the many strengths of this study, it is necessary to point out its limitations, which could be addressed in future research. In particular, the disproportionate nature of the sample could be included in the limitations of this study. Thus, there was a higher proportion of females and the social status of students (younger participants). However, this limitation need not be considered disruptive to the results and value of knowledge. The analysis was performed in the decomposition of identifiers, thus the problem of disproportionality of the sample was dispersed. Also, it must be emphasized that self-care is not the only factor in depression. Thus, the results should not be considered the only right pathway. Future research should address these limitations. Another limitation could be the fact that the SASS-14 measure is a new instrument and the factor of IICS showed relatively lower reliability values, which were accepted by the authors of the instrument. Therefore, future research should focus on this factor in order to find out whether it would show relatively low reliability also in other population groups. Regarding the limitations of the used models, it should be noted that causality was not examined in this study. For this reason, the findings cannot be interpreted as causal. All the results can only be understood in terms of associations, while a consideration of causal relationships can be misleading.

## Conclusion

The aim of the presented study was to evaluate the associations between self-care activities and depression in the general Slovak population, but also in its individual gender and age categories. The study answered the question how self-care activities are associated with depression. This provided a deeper insight into the issue, and the main findings support the general idea that well-practiced self-care activities should be an immediate part of an individual's life in order to improve mental health, especially to reduce depressive symptoms. In this context, SLP plays an important role, while HC indicates the need for increased attention during the pandemic. Public health efforts should focus on improving SLP and alleviating disturbing information that could adversely affect HC, and these efforts could be reflected in reducing depression. In this way, health literacy should be improved in Slovakia. Other dimensions of self-care have also shown significant results that should be taken into account. In terms of poor mental health, females and younger individuals need targeted interventions in this country. The findings call for immediate support for self-care behavior and the development of successful strategies aimed at the non-patient population. Slovak health policy leaders should focus on health-promoting preventive self-care interventions, as the demand for them increases even more during the pandemic. Gender and age characteristics should also be taken into account in this effort.

## Data Availability Statement

The raw data supporting the conclusions of this article will be made available by the authors, without undue reservation.

## Ethics Statement

The study was conducted according to the guidelines of the Declaration of Helsinki. The research was approved by the Ethics Committee of the Clinical Trials Services, USP TECHNICOM, Technical University of Košice, Slovakia (Ref. 02/03/2021 IG Bioinformatics). All participants included in the research confirmed their informed consent. The patients/participants provided their written informed consent to participate in this study.

## Author Contributions

BG: conceptualization, investigation, resources, writing—original draft preparation, visualization, writing—review and editing, supervision, project administration, and funding acquisition. BP: conceptualization, visualization, writing—review and editing, supervision, project administration, and funding acquisition. VI: conceptualization, investigation, resources, writing—original draft preparation, visualization, writing—review and editing, and supervision. MR: conceptualization, methodology, formal analysis, investigation, data curation, writing—original draft preparation, and writing—review and editing. All authors contributed to manuscript revision, read, and approved the submitted version.

## Funding

This research was supported by the Internal Grant Agency of FaME Tomas Bata University in Zlin: RO/2020/05: Economic quantification of marketing processes that focus on value increase for a patient in a process of system creation to measure and control efficiency in health facilities in the Czech Republic. This research was also supported by the Scientific Grant Agency of the Ministry of Education, Science, Research, and Sport of the Slovak Republic and the Slovak Academy Sciences as part of the research project VEGA 1/0797/20: Quantification of Environmental Burden Impacts of the Slovak Regions on Health, Social and Economic System of the Slovak Republic.

## Conflict of Interest

The authors declare that the research was conducted in the absence of any commercial or financial relationships that could be construed as a potential conflict of interest.

## Publisher's Note

All claims expressed in this article are solely those of the authors and do not necessarily represent those of their affiliated organizations, or those of the publisher, the editors and the reviewers. Any product that may be evaluated in this article, or claim that may be made by its manufacturer, is not guaranteed or endorsed by the publisher.
